# Measurement Modeling and Performance Analysis of a Bionic Polarimetric Imaging Navigation Sensor Using Rayleigh Scattering to Generate Scattered Sunlight

**DOI:** 10.3390/s24020498

**Published:** 2024-01-13

**Authors:** Zhenhua Wan, Kaichun Zhao, Haoyuan Cheng, Peng Fu

**Affiliations:** 1 School of Mechanical Engineering, Guangxi University, Nanning 530004, China; wanzh@gxu.edu.cn; 2 Department of Precision Instrument, Tsinghua University, Beijing 100084, China; fupeng2000@yeah.net; 3 College of Engineering, Ocean University of China, Qingdao 266100, China; chenghaoyuan@ouc.edu.cn

**Keywords:** polarimetric imaging, polarization navigation, Rayleigh scattering, bionic polarization, measurement error model

## Abstract

The bionic polarimetric imaging navigation sensor (BPINS) is a navigation sensor that provides absolute heading, and it is of practical engineering significance to model the measurement error of BPINS. The existing BPINSs are still modeled using photodiode-based measurements rather than imaging measurements and are not modeled systematically enough. This paper proposes a measurement performance analysis method of BPINS that takes into account the geometric and polarization errors of the optical system. Firstly, the key error factors affecting the overall measurement performance of BPINS are investigated, and the Stokes vector-based measurement error model of BPINS is introduced. Secondly, based on its measurement error model, the effect of the error source on the measurement performance of BPINS is quantitatively analyzed using Rayleigh scattering to generate scattered sunlight as a known incident light source. The numerical results show that in angle of E-vector (AoE) measurement, the coordinate deviation of the principal point has a greater impact, followed by grayscale response inconsistency of CMOS and integration angle error of micro-polarization array, and finally lens attenuation; in degree of linear polarization (DoLP) measurement, the grayscale response inconsistency of CMOS has a more significant impact. This finding can accurately guide the subsequent calibration of BPINS, and the quantitative results provide an important theoretical reference for its optimal design.

## 1. Introduction

Polarization is another dimension of light, just like spectrum and intensity, which can provide distinct and useful information about a visual scene [1] and is applied in many scenarios, such as microscopy imaging [2], optical precision measurement [3,4], and biological polarization navigation [5,6,7]. Many animals, particularly insects, are sensitive to the polarization of light and use this information for navigation, detection, and communication [8]. A great deal of research has been carried out on the behavioral neurobiology of polarization navigation in insects [9,10,11,12,13]. The desert ant has to rely heavily on polarized skylight and path integration in its featureless desert habitat during foraging [14]. Biologists have recently demonstrated that greater mouse-eared bats use skylight polarization cues to calibrate a magnetic compass at sunset [15] and mantis shrimp use celestial polarization and path integration to navigate home [16]. This celestial polarization orientation method is used as a bio-inspired polarization navigation method which has attracted much attention due to its advantages, namely autonomy and no error accumulation [17,18]. This method is reported to have potential applications in assisting inertial navigation in the event of satellite denial [19]. However, the factors that affect the measurement performance of bio-inspired polarization navigation sensor (BPNS) remain unclear. The study of the measurement error model of BPNS is of great significance to promote the practical application of polarization navigation.

Due to the different measurement principles of polarized skylight, the current measurement error models for BPNS are divided into photodiode based and polarimetric imaging based. In terms of the photodiode-based principle, Lambrinos et al. [20] designed a photodiode-based six-channel polarization compass for ground robot navigation and proved the feasibility of using polarized skylight for navigation. Later, Chu et al. [21] built an improved version of photodiode-based BPNS and investigated the dark noise and static sensitivity of this BPNS. Ma et al. [22] studied the inconsistent amplification of six photodiodes and the misalignment angle error of polarizers and used the NSGA-II algorithm to calibrate a BPNS similar to Chu’s. Wang et al. [23] presented an improved photodiode-based BPNS with a planoconvex lens, and used central-symmetry and a non-continuous calibration method. Chahl et al. [24] imitated the optical stabilization organ of dragonflies, known as the ocelli, and also developed a photodiode-based six-channel BPNS. Dupeyroux et al. [25,26,27] designed a photodiode-based BPNS, which can measure ultraviolet (UV) light, conducted outdoor performance tests under various weather conditions, and achieved an accuracy of 0.3° in clear sky. However, due to the fact that these photodiode-based BPNSs can only measure the polarization information at a certain point in the sky, they are susceptible to external factors such as weather interference and surrounding occlusion, resulting in poor robustness. To improve the robustness of the polarization navigation method, researchers have developed several bionic polarimetric imaging navigation sensors (BPINS). Sturzl et al. [28] performed a geometric calibration of BPINS based on four fisheye cameras and proposed an efficient method for reconstructing the full-sky polarization pattern. Liu et al. [29] used nanoimprint lithography to integrate a multi-directional nanowire grid polarizer on a complementary metal oxide semiconductor (CMOS) sensor to eliminate the installation angle error of the polarizer and conducted laboratory calibration. Fan et al. [30] analyzed the inconsistent response error of the CMOS and the installation angle error of the four polarizers and proposed a calibration method for a four-camera polarimetric imaging navigation device. Ren et al. [31] has reported a measurement model of BPINS based on the photodiode principle which takes into account the extinction ratio errors. Although the imaging prototypes are used for perception, the modeling and calibration methods of the photodiode-based principle are still used, and the Mueller matrix error of an optical system is not considered [29,30,31,32]. For models based on the principle of polarimetric imaging, the geometric errors of BPINS, such as principal point and lens distortion, are not considered [33,34,35,36], especially for the measurement of skylight. Our previous work only considered the angle error model for polarization sensors, and did not consider the overall measurement performance of the polarization sensor, which also includes the degree of linear polarization [37]. Polarized skylight imaging measurements involve the coordinate transformations and polarization state calculation of skylight, and BPINS measurements should be considered in terms of the geometric and polarization parameters of the optical system, which are not considered in the current research work [38,39,40,41,42].

Motivated by this situation, this article presents an error model for the measurement process of BPINS considering the geometrical and polarization errors of the optical system. Using Rayleigh scattering to generate the skylight with a known polarization state, an analysis method is proposed for the effect of error sources on the overall measurement performance of BPINS. The proposed analysis method can validate the overall measurement performance of all BPINSs. The contributions of this paper can be concluded as follows:

(1) An analysis method is proposed to analyze the influence of key error sources on the measurement performance of BPINS, including the influence of the degree of linear polarization and angle of E-vector. The proposed analysis method is generalized for all BPINSs.
(2) The key error factors affecting the measurement accuracy of BPINS are quantitatively investigated using a skylight with a known polarization state generated by Rayleigh scattering as the incident light source, which is similar to the polarization pattern of an outdoor Rayleigh clear sky.
(3) This work can guide the calibration of BPINS and provide a theoretical basis for the optimal design of BPINS. In addition to BPINS, the idea of this work can be applied to other polarimetric imaging applications such as polarimetric underwater detection, polarimetric defogging, polarimetric medical diagnostics, and so on.

The remainder of this paper is organized as follows: A measurement principle and error model of BPINS are presented in Section 2. A detailed description of the proposed performance analysis method of BPINS is presented in Section 3. The conclusions are presented in Section 4.

## 2. Measurement Principle and Error Model of BPINS

### 2.1. Principle of Skylight Polarimetric Imaging

**Figure 1:**
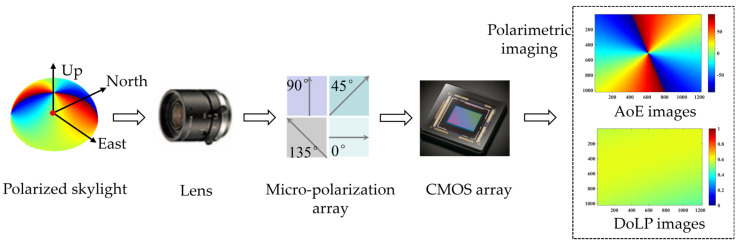
Measurement process of the bio−inspired polarimetric imaging navigation sensor.

Sunlight is scattered through the atmosphere and forms polarized light in the sky. To clearly understand the factors that affect the measurement accuracy of BPINS, we need to model the measurement errors of BPINS. We analyze the whole measurement process of imaging skylight into the CMOS plane. Firstly, the polarized skylight under the local geographical system passes through multiple lenses inside the lens and is focused at the exit pupil; then it passes through the micro-polarization array and is finally imaged into the CMOS plane. The measurement process of BPINS is shown in Figure 1.

**Figure 2:**
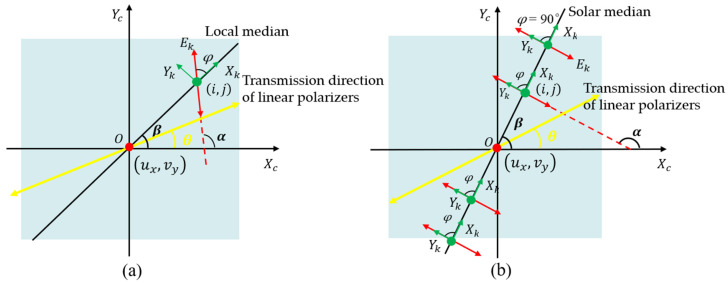
Projection of polarized light from the sky (in the local coordinate system) on the imaging plane; (**a**) the projection of any incident light beam; (**b**) the projection of the incident light beam on the solar meridian.

The E-vector of the polarized skylight is a three-dimensional representation in the east–north–up coordinate system. Skylight polarimetric imaging is the projection of each beam of polarized skylight in the field of view onto the CMOS imaging plane through the Muller matrix of the optical system of BPINS. Figure 2 shows the projection process of two-dimensional polarized skylight from a local coordinate system in the BPINS coordinate system. The Stokes vector can represent both fully and partially polarized light. Since polarized skylight is partially polarized light, the Stokes vector for polarized skylight can be expressed as follows:

(1) $$S_{skylight}=\left[\begin{array}{c} I\\ Q\\ U\\ V \end{array}\right]=I\left[\begin{array}{c} 1\\ d\cos2\chi \cos2\psi \\ d\cos2\chi \sin2\psi \\ d\sin2\chi \end{array}\right]$$

where the I parameter is the total intensity of the skylight; the Q parameter describes the amount of linear horizontal or vertical polarization; the parameter U is the amount of linear +45° or −45° polarization. The parameter V is the amount of right or left circular polarization contained within the beam, and the content of this component in the skylight is extremely small [1], so it can be ignored in the skylight imaging. The parameter d is the degree of polarization of skylight; φ represents the angle of polarization of skylight; χ is the ellipticity angle of skylight.

According to the principle of polarized skylight imaging, polarized skylight imaging is the process of projecting each beam of polarized skylight in the field of view onto the imaging plane through the Mueller matrix of the optical system. The Stokes vector of the outgoing light is as follows:

(2) $$S_{out}=M_{\theta }\cdot M_{lens}\cdot S_{skylight}$$

where the incident polarized skylight is $S_{skylight}$ , the Mueller matrix of the lens is $M_{lens}$ (if the error is not taken into account, $M_{lens}$ is the unit matrix), and the Mueller matrix of the micro-polarizer with azimuth θ is $M_{\theta }$ . The Stokes vector of the outgoing light is $S_{out}$ after passing through the Mueller matrix of an optical system.

Since the CMOS of BPINS can only perceive the total intensity of the Stokes vector of skylight, the general equation for the total intensity received by the CMOS imaging plane is as follows:

(3) $$I_{\theta }=\frac{1}{2}(I+Q\cos2\theta +U\sin2\theta )$$

Taking θ=0°,45°,90° into Equation (3), a linear system of equations can be obtained as follows:

(4) $$\left[\begin{array}{c} I_{0}\\ I_{45}\\ I_{90} \end{array}\right]=\frac{1}{2}\left[\begin{array}{ccc} 1 & 1 & 0\\ 1 & 0 & 1\\ 1 & -1 & 0 \end{array}\right]\left[\begin{array}{c} I\\ Q\\ U \end{array}\right]$$

According to the definition of the angle of polarization and degree of linear polarization (DoLP), the formulae for calculating the angle of polarization and DoLP can be obtained as follows:

(5) $$\left\{\begin{array}{c} \tan2\alpha =\frac{U}{Q}\\ d=\frac{\sqrt{U^{2}+Q^{2}}}{I} \end{array}\right.$$

Based on the definition of the angle of E-vector (AoE) for polarized skylight which is the angle between the direction of E vector vibration and the local meridian, the angle of E-vector in the CMOS imaging plane can be solved as follows:

(6) $$\left\{\begin{array}{c} \varphi =\alpha -\beta ,\left(-90^{\circ }<\varphi \leq 90^{\circ }\right)\\ \alpha =\frac{1}{2}\arctan\frac{\left(2I_{45}-I_{0}-I_{90}\right)}{\left(I_{0}-I_{90}\right)}\\ \beta =\arctan\frac{\left(j-v_{y}\right)}{\left(i-u_{x}\right)} \end{array}\right.$$

where φ represents the angle of E-vector, α is the angular distances of E-vector from the reference axis $X_{c}$ of BPINS, and β represents the azimuth angle of the local meridian from the reference axis $X_{c}$ of BPINS. $\left(i,j\right)$ are the coordinates of the incident skylight; $\left(u_{x},v_{y}\right)$ is the principal point of the AoE image.

### 2.2. Measurement Error Model of BPINS

From the above Equation (6), it can be seen that the factors affecting the polarimetric measurement accuracy of the BPINS mainly include the coordinate offset of principal point, the integration angle error of the micro-polarization array, the lens attenuation, and the grayscale response inconsistency of CMOS. The continuous form of the measurement error model for BPINS is noted as:

(7) $$\varphi \left(\delta I_{\theta },\delta \beta ,\delta \theta ,\delta \rho \right)=\frac{\partial f}{\partial I_{\theta }}\delta I_{\theta }+\frac{\partial f}{\partial \beta }\delta \beta +\frac{\partial f}{\partial \theta }\delta \theta +\frac{\partial f}{\partial \rho }\delta \rho$$

where $\delta I_{\theta },\delta \beta ,\delta \theta ,\delta \rho$ , respectively, represent the inconsistent grayscale response of CMOS, the coordinate deviation of principal point, the installation angle error of micro-polarization array, and the lens attenuation.

(1) Coordinate deviation of principal point

**Figure 3:**
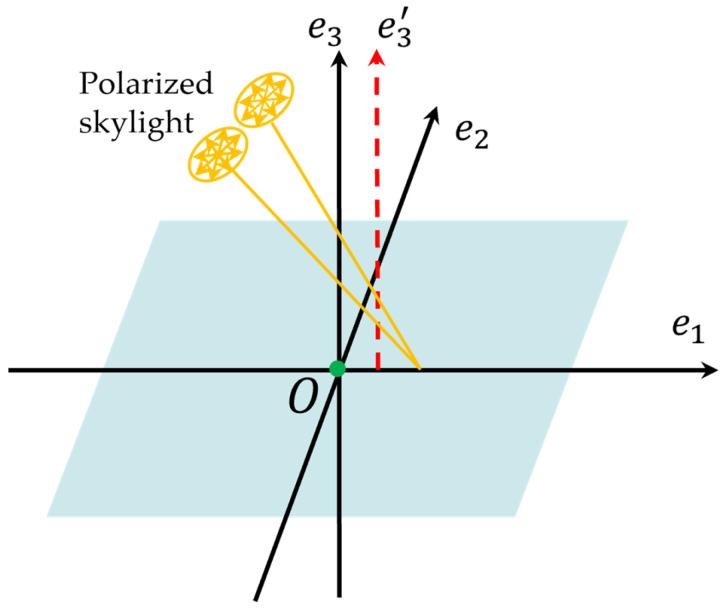
The influence of the coordinates offset of the principal point.

When the incident skylight is coplanar with the direction of the maximum offset of the principal point coordinates, the influence form of the coordinates’ deviation from the principal point is shown in Figure 3, assuming that $e_{1}$ is along the direction of the maximum offset of the principal point coordinates, $e_{3}$ is the direction of the ideal principal optical axis, and $e_{3}^{\prime }$ is the direction of the actual principal optical axis. Since the image principal point of BPINS is not calibrated, the actual principal point coordinate position is offset from the ideal principal point coordinate position, i.e., there is an error in $\left(u_{x},v_{y}\right)$ in β . In the case that other error variables are zero, the effect of the coordinate deviation of the principal point on the AoE image can be obtained from Equations (6) and (7) as follows:

(8) $$\left\{\begin{array}{c} \varphi =\alpha -\beta \\ \beta =\arctan\left(\frac{j-v_{y}}{i-u_{x}}+\delta \beta \right),\delta \beta =\frac{\delta u_{x}\left(j-v_{y}\right)-\delta v_{y}\left(i-u_{x}\right)}{\left(j-v_{y}-\delta v_{y}\right)\left(j-v_{y}\right)} \end{array}\right.$$

where $\left(\delta u_{x},\delta v_{y}\right)$ is the coordinate deviation of the principal point.

(2) Installation angle error of micro-polarization array

**Figure 4:**
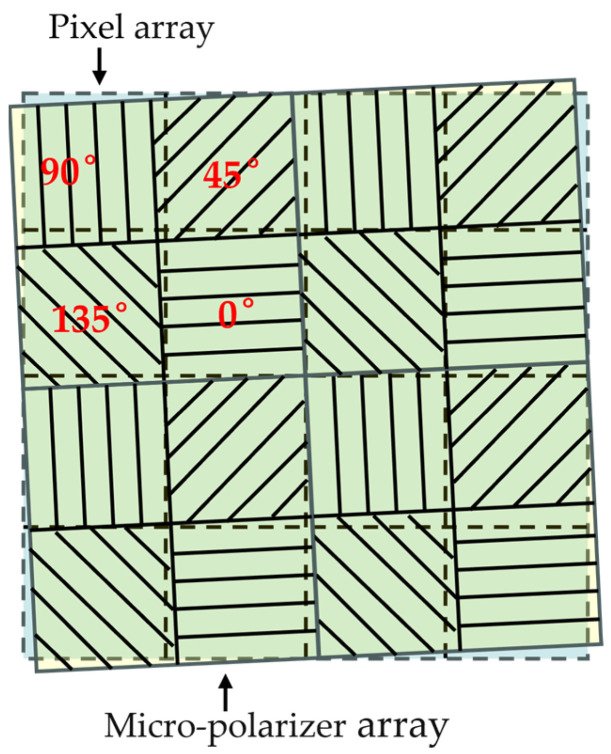
Installation angle error of micro-polarizer array.

The incident polarized beam interacts with the micro-polarization array after passing through the lens. The installation angle error of the micro-polarization array has an effect on the Mueller matrix of the micro-polarization array, as shown in Figure 4, the Mueller matrix of the micro-polarization array with an azimuth of θ including the installation angle error δθ can be written as:

(9) $$M_{\theta }=\frac{1}{2}\left[\begin{array}{ccc} 1 & \cos2\left(\theta +\delta \theta \right) & \sin2\left(\theta +\delta \theta \right)\\ \cos2\left(\theta +\delta \theta \right) & \cos^{2}2\left(\theta +\delta \theta \right) & \sin2\left(\theta +\delta \theta \right)\cos2\left(\theta +\delta \theta \right)\\ \sin2\left(\theta +\delta \theta \right) & \sin2\left(\theta +\delta \theta \right)\cos2\left(\theta +\delta \theta \right) & \sin^{2}2\left(\theta +\delta \theta \right) \end{array}\right]$$

(3) Lens attenuation

When the polarized skylight passes through the lens, the incident skylight beam corresponding to a certain pixel can be decomposed into mutually orthogonal p light and s light. Let $\tau_{1}$ and $\tau_{2}$ indicate the amplitude transmittance of the lens to p light and s light, respectively, which only varies with the angle of incidence.

Each beam of incident skylight has a different angle of incidence with each optical surface and its azimuthal angle of the incident surface, which has a certain symmetry. If a pixel corresponds to a beam of incident skylight, then the Mueller matrix of the lens micro-unit corresponding to a single pixel in the local coordinate system can be expressed as:

(10) $$M_{lens}=\frac{\tau_{1}^{2}+\tau_{2}^{2}}{2}\left[\begin{array}{ccc} 1 & \rho & 0\\ \rho & 1 & 0\\ 0 & 0 & \sqrt{1-\rho^{2}} \end{array}\right],\rho =\frac{\tau_{1}^{2}-\tau_{2}^{2}}{\tau_{1}^{2}+\tau_{2}^{2}}$$

where ρ denotes the linear bidirectional attenuation of the lens.

(4) Inconsistency of CMOS grayscale response

In visual measurement, there is an approximate linear relationship between input light intensity and image grayscale. The nonuniformity of CMOS grayscale response mainly consists of the nonuniformity of the dark current response and photoelectric response. The photoelectric sensitive array outputs the light intensity $I_{\theta }$ as image grayscale. Based on the linear model in EMVA 1288 [43], the grayscale of the CMOS output can be expressed as:

(11) $${\tilde{I}}_{\theta }=aI_{\theta }+b_{\theta }+n_{\theta },\left(\theta =0{}^{\circ},45{}^{\circ},90{}^{\circ}\right)$$

According to the principle of polarized skylight imaging, the CMOS of BPINS can only perceive the total intensity of the Stokes vector of skylight. From Equation (2), the general equation for the total intensity received by the CMOS imaging plane can be written as follows:

(12) $${\tilde{I}}_{\theta }=\frac{\tau_{1}^{2}+\tau_{2}^{2}}{4}\left(\tilde{I}\left(1+\rho \cos2\left(\theta +\delta \theta -\beta \right)\right)+Q\left(\rho +\cos2\left(\theta +\delta \theta -\beta \right)\right)+U\sin2\left(\theta +\delta \theta -\beta \right)\cdot \sqrt{1-\rho^{2}}\right)$$

Taking θ=0°,45°,90° into Equation (12), $\tilde{I},Q,U$ can be solved as follows:

(13) $$\left\{\begin{array}{c} \tilde{I}=-\frac{2}{\left(\rho^{2}-1\right)\left(\tau_{1}^{2}+\tau_{2}^{2}\right)}\left({\tilde{I}}_{0}\left(1-\rho \left(\cos2\left(\delta \theta +\beta \right)+\sin2\left(\delta \theta +\beta \right)\right)\right)+{\tilde{I}}_{90}\left(1+\rho \left(\cos2\left(\delta \theta +\beta \right)+\sin2\left(\delta \theta +\beta \right)\right)\right)-2{\tilde{I}}_{45}\rho \sin2\left(\delta \theta +\beta \right)\right)\\ Q=\frac{2}{\left(\rho^{2}-1\right)\left(\tau_{1}^{2}+\tau_{2}^{2}\right)}\left({\tilde{I}}_{0}\left(\sin2\left(\delta \theta +\beta \right)-\cos2\left(\delta \theta +\beta \right)+\rho \right)+{\tilde{I}}_{90}\left(\cos2\left(\delta \theta +\beta \right)+\sin2\left(\delta \theta +\beta \right)+\rho \right)-2{\tilde{I}}_{45}\sin2\left(\delta \theta +\beta \right)\right)\\ U=-\frac{2}{\sqrt{1-\rho^{2}}\left(\tau_{1}^{2}+\tau_{2}^{2}\right)}\left({\tilde{I}}_{0}\left(\cos2\left(\delta \theta +\beta \right)+\sin2\left(\delta \theta +\beta \right)\right)+{\tilde{I}}_{90}\left(\cos2\left(\delta \theta +\beta \right)-\sin2\left(\delta \theta +\beta \right)\right)-2{\tilde{I}}_{45}\cos2\left(\delta \theta +\beta \right)\right) \end{array}\right.$$

Based on the definition of the angle of E-vector and the degree of linear polarization of skylight, the polarimetric parameters of BPINS can be obtained as:

(14) $$\left\{\begin{array}{c} \tan2\varphi =\frac{U}{Q}\\ d=\frac{\sqrt{U^{2}+Q^{2}}}{\tilde{I}} \end{array}\right.$$

The measurement model of BPINS with measurement error can be written as a function expressed as:

(15) $$\left\{\begin{array}{c} \varphi =f\left(I_{\theta },\beta ,\theta ,\rho \right)\\ d=g\left(I_{\theta },\beta ,\theta ,\rho \right) \end{array}\right.$$

## 3. Performance Analysis Method for Generating Sunlight Using Rayleigh Scattering

As seen in Section 2, outdoor polarized skylight is produced by the scattering of direct sunlight through the atmosphere. It is not possible to fully generate polarized skylight indoors, but we can obtain outdoor polarized skylight using the sky Rayleigh scattering model and the polarimetric imaging principle. In this section, the outdoor polarized skylights were numerically simulated for known incident light, and then we analyzed the extent to which each error source affects the measurement performance of BPINS. 

Once the solar position is known in the local geographical coordinate system, the skylight polarization distribution pattern can be established. To truly simulate the polarized skylight field at a certain time on a certain day, numerical simulations are performed at a solar altitude angle of 5° and a solar azimuth angle between 10° and 80° to obtain the skylight polarization pattern formed at different positions of the sun. The true values of the AoE image and the DoLP image can be obtained by the Rayleigh scattering model. The two parameters of the AoE and DoLP can be used to characterize the polarization state of each beam of polarized skylight by Poincare sphere. Any point on the equator of the Poincare sphere represents completely linearly polarized light in different directions. Since the polarized skylight is partially linearly polarized light, its maximum DoLP should be less than 1. According to the conversion relation between the Poincare sphere and the Stokes vector, we can get the Stokes vector of each beam of polarized skylight, and thus obtain the known polarization state of the skylight.

**Figure 5:**
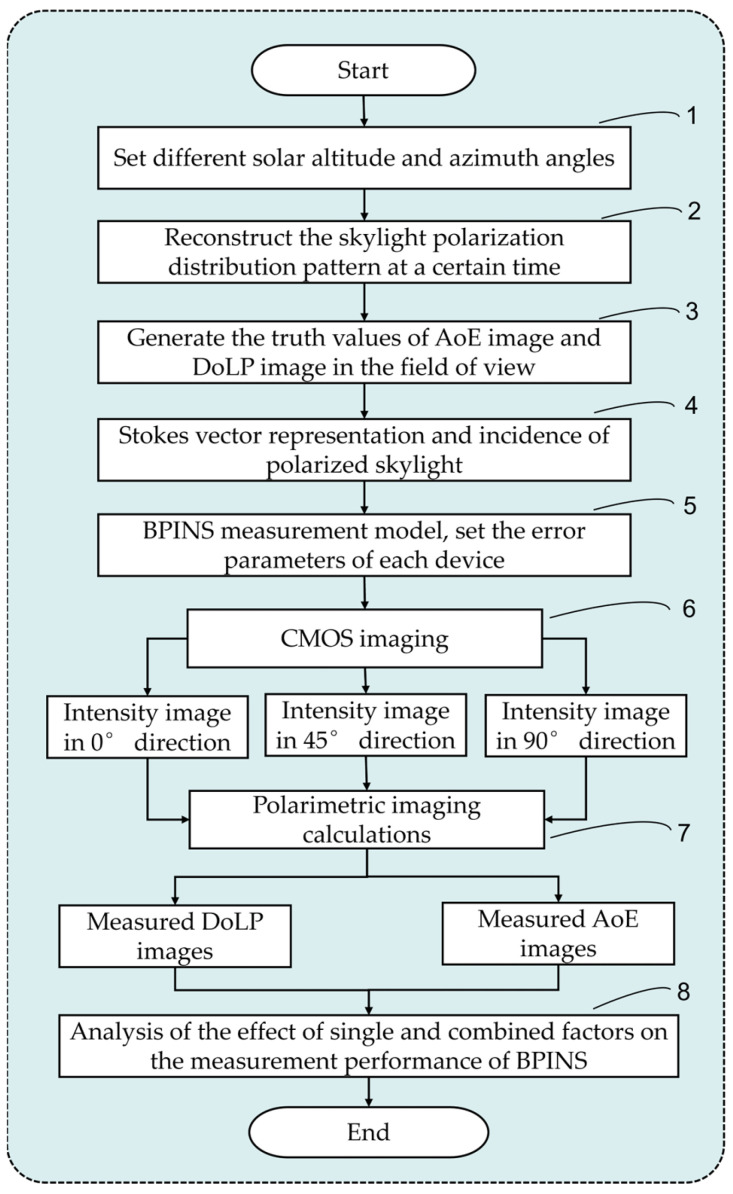
Flow chart of the impact analysis of the measurement error model of BPINS.

The skylight in a known polarization state is incident on the BPINS, which interacts with the micro-polarization array through the lens and is finally imaged on the CMOS plane of the BPINS. Based on the measurement error model described above, the skylight polarimetric imaging calculation is performed by sequentially varying each error source to form 0°, 45°, and 90° intensity images, which are then calculated to obtain the AoE images and DoLP images. Figure 5 shows the impact analysis flowchart of the measurement error model of BPINS. The specific steps are as follows:

1. Set different solar altitude and azimuth angles. We set the solar altitude angle to 5° during the simulation, and the solar azimuth angle to change every 10° between 10° and 80°.
2. Reconstruct the skylight polarization distribution pattern at a certain time, and the skylight polarization distribution pattern can be reconstructed in the local geographical coordinate system according to the set solar altitude angle and azimuth angle and Rayleigh scattering model.
3. Generate the truth values of the AoE image and DoLP image in the field of view. According to the theoretical BPINS projection model, CMOS and lens parameters, we can get the truth values of the two-dimensional AoE image and DoLP image from the three-dimensional skylight polarization distribution pattern reconstructed in step 2.
4. Stokes vector representation and incidence of polarized skylight. The polarization state of incident light can be calculated from the truth value of the two-dimensional AoE image and DoLP image obtained in step 3, and the polarization state of incident light is represented by the Stokes vector.
5. BPINS measurement model, set the error parameters of each device (Table 1), and polarized skylight with a known polarization state obtained from step 4 is incident into BPINS with measurement errors.
6. CMOS imaging. Through the BPINS projection model, CMOS and lens parameters, we can get 0°, 45°, 90° direction intensity images.
7. Polarimetric imaging calculation. The intensity images obtained in step 6 are used for polarimetric imaging calculation to obtain DoLP and AoE images containing measurement errors.
8. Analyze the effect of single and combined factors on the measurement performance of BPINS. Repeat the above steps 1–7 to obtain multiple sets of DoLP and AoE images containing measurement errors, and then analyze the influence of errors on the measurement performance of BPINS.

Due to the need to conduct multiple numerical simulation experiments, it is necessary to simulate the distribution of each error source. Here, the distribution of each error source is set as Gaussian distribution, and the specific simulation parameters of each error source are set as shown in Table 1. To reduce the computational time, the image resolution of BPINS is set to (1024, 1224) pixels, and the maximum DoLP is set to 0.6, which corresponds to a maximum greyscale response of 153 (DN), due to cloud cover and light intensity attenuation during the transmission of polarized skylight. Based on the position of the origin of the image coordinate system in the pixel coordinate system, the ideal principal point is (512.5, 612.5) pixels, and the specific parameters of BPINS are set as shown in Table 2. We then used Monte Carlo [44] methods to analyze the effects of single and combined factors on the measurement performance of BPINS, respectively. 

## 4. Numerical Results

### 4.1. Effect of a Single Factor on the Measurement Performance of BPINS

**Figure 6:**
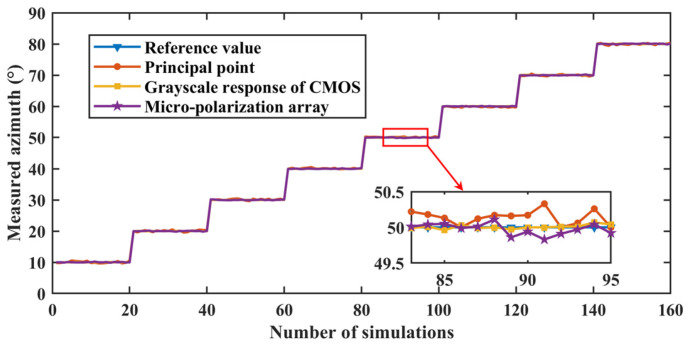
Azimuth measurement results for the effect of a single factor.

**Figure 7:**
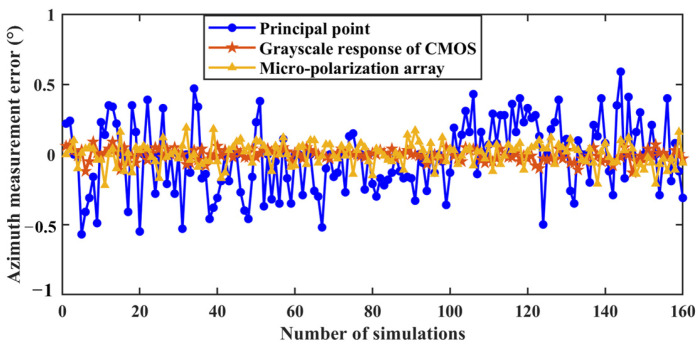
Azimuth measurement errors corresponding to a single factor.

According to the error parameter setting in Table 1 and the measurement model of BPINS, 160 numerical simulation experiments were conducted randomly for each error source, and the solar azimuth angle was changed every 20 times. Eight sets of numerical simulation experiments were performed at a solar altitude angle of 5° and a solar azimuth angle between 10° and 80° to obtain the skylight polarization pattern. We started the first set of experiments setting the solar azimuth angle to 10° and conducted one set of experiments for every 10° increase. The set solar azimuth angle is reciprocal to the measured azimuth angle, which adds up to 90°. The reference value of the azimuth is 90° minus the set solar azimuth. The simulated skylight with a known polarization state is incident into the measurement model of BPINS, and the azimuth angle is extracted from the AoE image with the measurement error, and the measured azimuth is compared with the ideal azimuth. Figure 6 shows azimuth measurement results for the effect of a single factor. Figure 7 shows the azimuth measurement errors corresponding to a single factor. 

Table 3 shows the results of the quantitative azimuth measurement errors for each error source. As can be seen from Table 3, the azimuth measurement error is 0.2476° for the condition that the coordinate offset of a principal point satisfies the $\delta \beta ∼N\left(0,2^{2}\right)$ distribution.

**Figure 8:**
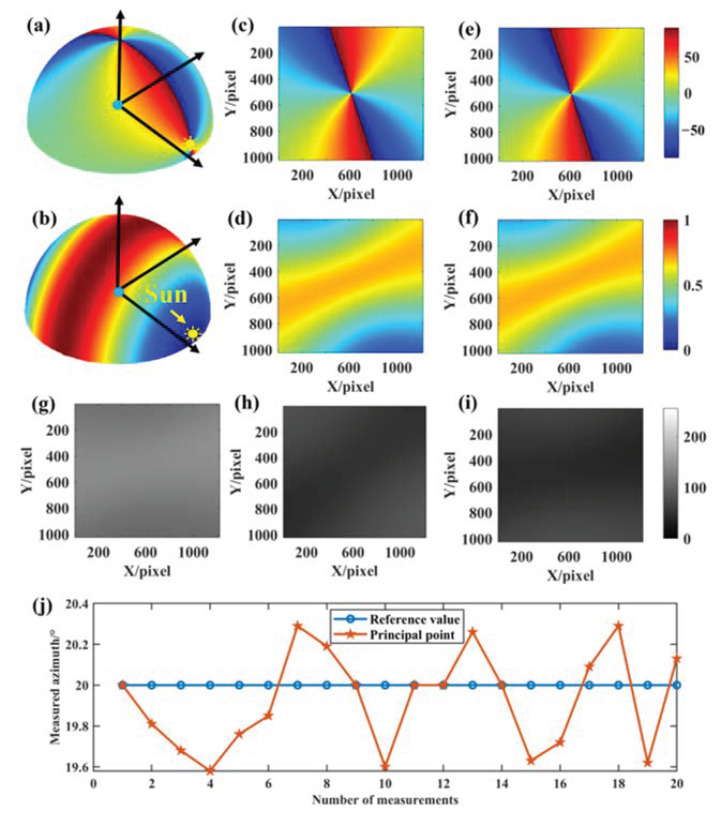
Imaging results of coordinate offset of a principal point. (**a**,**b**) are the three-dimensional skylight polarization pattern for a solar altitude angle of 5° and an azimuth angle of 70°; (**c**) the true AoE image; (**d**) the true DoLP image; (**e**) the measured AoE image; (**f**) the measured DoLP image; (**g**–**i**) are the 0°, 45°, and 90° directional intensity images, respectively; (**j**) is the result of azimuth extraction for 20 simulations with the solar azimuth set to 70°, where the reference value is 20° and the measured value is reciprocal to the set solar azimuth.

Figure 8 shows an example of the imaging results obtained by simulating the coordinate offset of a principal point. Figure 8a,b show the three-dimensional skylight polarization pattern for a solar altitude angle of 5° and an azimuth angle of 70° in the local geographical system. Figure 8c,d show the true value of the AoE image and the true value of the DoLP image in the field of view, respectively. The Stokes vectors of scattered sunlight under the Rayleigh scattering sky can be obtained from Figure 8c,d. These scattered skylights are incident on the sensor to form the 0°, 45°, and 90° directional intensity images, as shown in Figure 8g–i. Figure 8e,f can be solved from the 0°, 45°, and 90° intensity images. Figure 8j shows the result of azimuth extraction for 20 simulations when the solar azimuth in the local geographic system is set to 70°, where the reference value is 20° and the measured azimuth values are reciprocal to the set solar azimuth.

**Figure 9:**
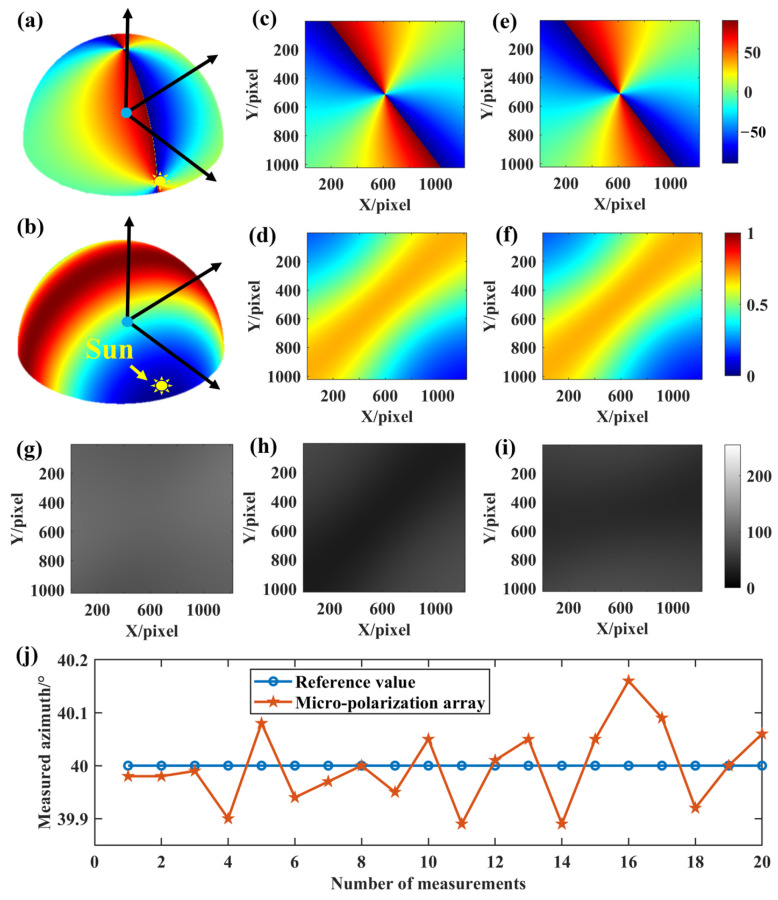
Imaging results of installation angle error of a micro-polarization array. (**a**,**b**) are the three-dimensional skylight polarization patterns for a solar altitude angle of 5° and an azimuth angle of 50°; (**c**) the true AoE image; (**d**) the true DoLP image; (**e**) the measured AoE image; (**f**) the measured DoLP image; (**g**–**i**) are the 0°, 45°, and 90° directional intensity images, respectively; (**j**) is the result of azimuth extraction for 20 simulations with the solar azimuth set to 50°.

Figure 9 shows an example of the experimental imaging results for the integration angle error of the micro-polarization array. Figure 9a,b show the three-dimensional skylight polarization pattern for a solar altitude angle of 5° and an azimuth angle of 50°. Figure 9c,d show the true value of the AoE image and the true value of the DoLP image in the field of view, respectively. The Stokes vectors of scattered sunlight under the Rayleigh scattering sky are obtained from Figure 9c,d. These scattered skylights are incident on the sensor to form the 0°, 45°, and 90° directional intensity images, as shown in Figure 9g–i. It can be seen that the measured AoE image, i.e., Figure 9e, is as smooth as the true value of the AoE image. The same is true for the measured DoLP image. As can be seen from Table 3, the azimuthal measurement error is 0.0812° under the condition that the integration angle error of the micro-polarization array satisfies the (0, $0.1^{2}$ ) distribution. This indicates that an integration angle error of 0.1° for the micro-polarization array has a relatively small effect on the polarization measurements of BPINS.

**Figure 10:**
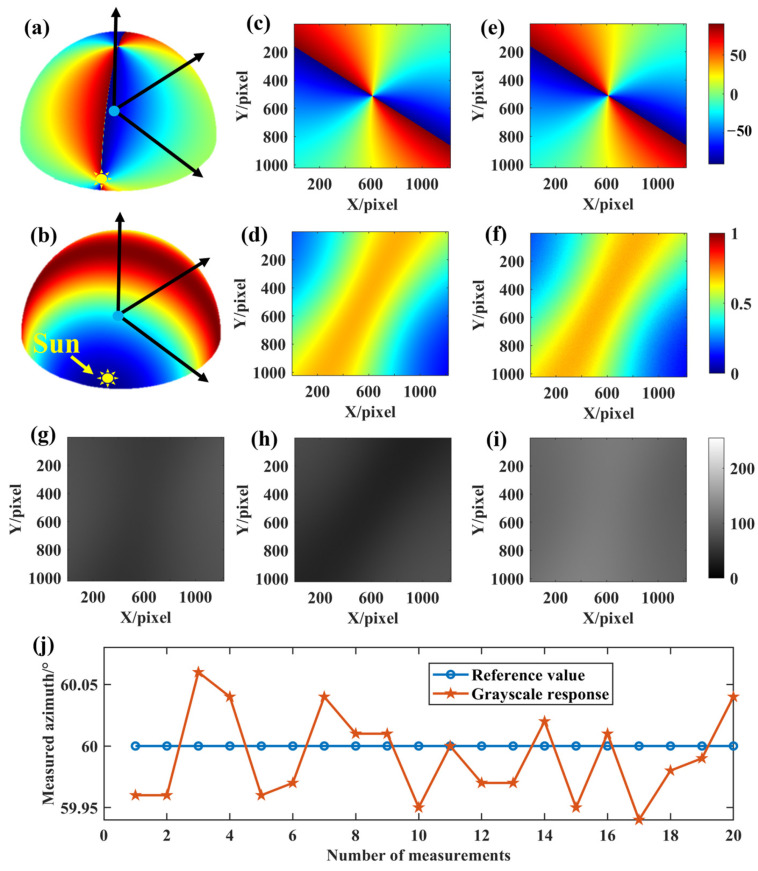
Imaging results of grayscale response inconsistent of CMOS. (**a**,**b**) show the three-dimensional skylight polarization patterns for a solar altitude angle of 5° and an azimuth angle of 30°; (**c**) the true AoE image; (**d**) the true DoLP image; (**e**) the measured AoE image; (**f**) the measured DoLP image; (**g**–**i**) are the 0°, 45°, and 90° directional intensity images, respectively; (**j**) is the result of azimuth extraction for 20 simulations with the solar azimuth set to 30°.

Figure 10 shows an example of the simulated experimental imaging results for the grayscale response inconsistency of CMOS. Figure 10a,b show the three-dimensional skylight polarization pattern for a solar altitude angle of 5° and an azimuth angle of 30°. Figure 10c,d show the true value of the AoE image and the true value of the DoLP image in the field of view, respectively. The Stokes vectors of scattered sunlight under the Rayleigh scattering sky are obtained from Figure 10c,d. These scattered skylights are incident on the sensor to form the 0°, 45°, and 90° directional intensity images, as shown in Figure 10g–i. Figure 10e shows that the measured AoE image is not as smooth as the true value, especially at the edges, and the same is true for the measured DoLP image. As can be seen from Table 3, the azimuth measurement error is 0.0405° with a mean error close to 0.0059° under the condition that the grayscale response inconsistency of CMOS satisfies the (0, $1^{2}$ ) distribution.

As can be seen from Table 3, when the bilinear attenuation of the lens is controlled to within (0.2, 0.2), the azimuthal measurement error is zero. This indicates that the bilinear attenuation of the lens has no effect on the AoE image, but has an effect on the DoLP image, as the DoLP is determined by the light intensity.

From the above numerical simulation results, the following conclusions can be drawn: the coordinate deviation of the principal point, the installation error of the micro-polarization array and the grayscale response inconsistency of CMOS are the important error sources that affect the azimuth measurement. These simulation results can guide the later calibration of BPINS.

### 4.2. Effect of Combined Factors on the Measurement Performance of BPINS

The simulation results in Section 4.1 provide a quantitative indication of the extent to which a single factor affects the measurement performance of BPINS. However, it is also necessary to analyze the combined effect of these four error sources on the measurement performance of BPINS. After all, the measurement accuracy of an uncalibrated BPINS is largely determined by the combination of these error sources.

**Figure 11:**
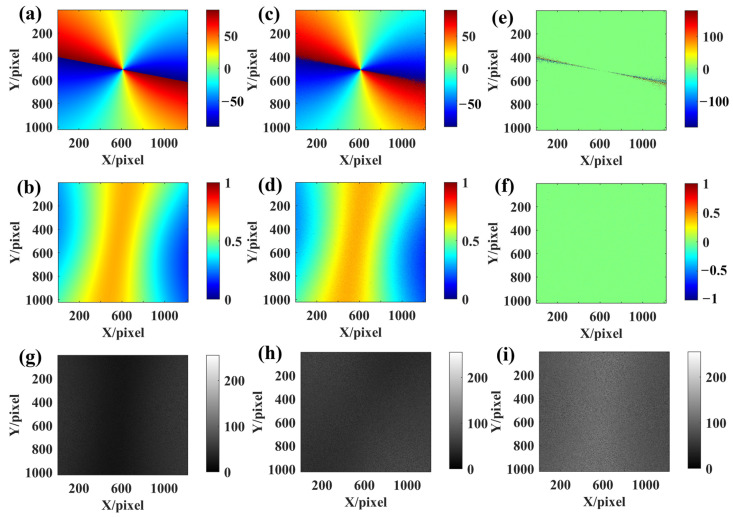
Imaging results of a comprehensive experiment for a solar altitude angle of 5° and an azimuth angle of 20°. (**a**) the true AoE image; (**b**) the true DoLP image; (**c**) the measured AoE image; (**d**) the measured DoLP image; (**e**) is the difference between the true AoE image and the measured AoE image; (**f**) the difference between the true DoLP image and the measured DoLP image; (**g**–**i**) are the 0°, 45°, and 90° directional intensity images, respectively.

**Figure 12:**
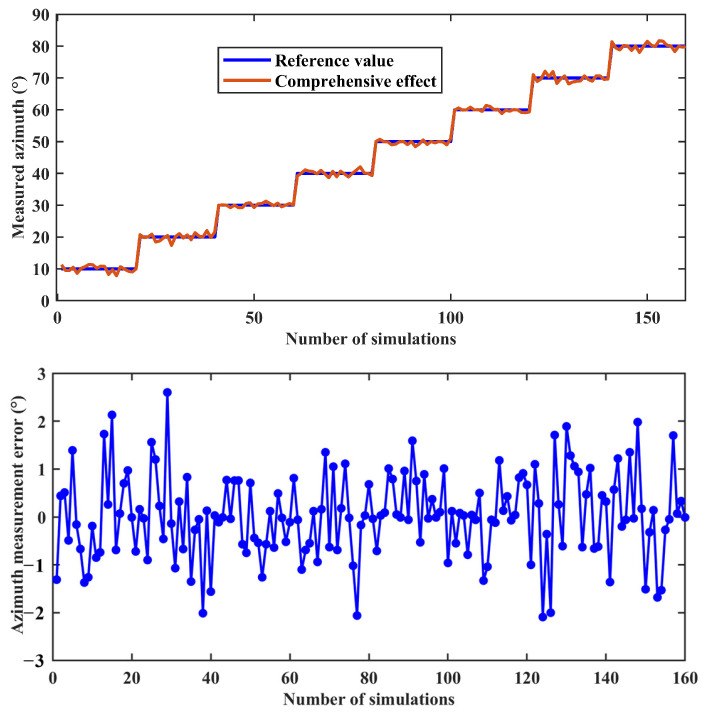
Azimuth measurement results affected by combined factors.

Combined simulation experiments were conducted with the same number of times as the single factors, also changing the solar azimuth every 20 times. Figure 11 shows an example of the imaging results from one of the comprehensive simulation experiments for a solar altitude angle of 5° and an azimuth angle of 20°. Figure 11a,b show the true AoE image and the true DoLP image, respectively, used to generate the Rayleigh scattered skylight incident into the uncalibrated BPINS. Figure 11c,d are the AoE image and DoLP image measured by the BPINS, and Figure 11e is the difference between the true AoE image and the measured AoE image. As can be seen from Figure 11c, the measured AoE image is not very smooth, especially at the edges, in the same way as the measured DoLP. Figure 12 shows the azimuth measurements affected by the combined effect of these four error sources. A total of 160 combined simulation experiments show that for uncalibrated BPINS, the combined effect of these error sources has a relatively large impact on the BPINS measurements. Under the combined influence of these error sources, the azimuth measurement accuracy of BPINS is 0.8839°.

Using the above Monte Carlo simulation, for the BPINS to be calibrated, the coordinate deviation of principal point is controlled to within (2, 2) pixels, with an azimuthal measurement error of 0.2476°. The integration angle error of micro-polarization array and grayscale response inconsistency of CMOS are controlled to within 0.1° and 1.0 (DN), respectively, with azimuthal measurement errors of 0.080° and 0.0456°, within the same order of magnitude. The lens attenuation is controlled to within (0.2, 0.2), and the azimuthal measurement error is zero, indicating that the light intensity attenuation has no effect on the AoE image. From the above numerical simulation results, the following conclusions can be drawn: the combined effects of the error sources have a relatively large impact on the polarization measurements of BPINS. The coordinate deviation of the principal point, the integration angle error of the micro-polarization array and the grayscale response inconsistency of CMOS are the key error sources to be considered for subsequent calibration of BPINS.

## 5. Conclusions

This paper systematically analyzes the measurement errors of skylight passing through the lens to the micro-polarization array and finally the incident on the CMOS imaging plane. A Stokes vector-based measurement model of BPINS is developed, taking into account multiple source factors, such as the principal point, the grayscale response of CMOS, the integration angle of the micro-polarization array, and the lens. We simulated the Rayleigh scattered skylight as a known incident light source, performed outdoor measurement model simulations, and quantitatively analyzed the extent to which the error sources affect the measurement performance of BPINS. The results show that the coordinate deviation of the principal point has a more significant impact on AoE measurement, followed by the grayscale response inconsistency of CMOS and integration angle error of micro-polarization array, and finally lens attenuation; in DoLP measurement, the grayscale response inconsistency of CMOS has a more significant impact. This finding can guide the subsequent calibration of BPINS, and the quantitative results provide an important reference basis for its optimization design and calibration experiments. The proposed error model and impact analysis method for BPINS can verify the measurement performance of all BPINSs.

In this paper, we just used the Rayleigh scattering model to simulate the outdoor polarization pattern of a clear sky and analyze the measurement performance of the polarimetric imaging navigation sensor. Since there are also complex scattering media such as haze or clouds in the outdoor sky, there are a lot of meaningful and important works on light interaction with complex or strong scattering media [45,46,47,48], such as Chen et al. [45,46]. These works are worth studying and referring to, and this is the focus of our next research.

## Figures and Tables

**Table 1:** Simulation parameter settings for error sources.

Error Sources	Distribution (*μ*, *σ*)	
	μ	σ
Coordinate deviation of principal point	0	(2, 2) pixel
Installation angle error of micro-polarization array	0	0.1°
Lens attenuation	0	(0.2, 0.2)
Grayscale response inconsistency of CMOS	0	1 (DN)

**Table 2:** Parameter settings of BPINS.

Parameter	Specific Value	Unit
Pixel size	3.45 × 3.45	μm
Image resolution	(1024, 1224)	pixel
Focus length	8	mm
Principal point	(512.5, 612.5)	pixel

**Table 3:** Experiments results of azimuth measurements.

Error Sources	Azimuth Measurement Error (*μ*, *σ*)	
	μ	σ
Coordinate deviation of principal point	−0.0237	0.2476
Installation angle error of micro-polarization array	0.0018	0.0812
Grayscale response inconsistency of CMOS	0.0059	0.0405
Lens attenuation	0.0	0.0

## Data Availability

The data that support the findings of this study are available from the corresponding author upon reasonable request.
